# Syntheses and crystal structures of three salts of 1-(4-nitro­phenyl)­piperazine

**DOI:** 10.1107/S205698902300302X

**Published:** 2023-04-06

**Authors:** Holehundi J. Shankara Prasad, Devaraju, Hemmige S. Yathirajan, Mehmet Akkurt, Sabine Foro, Rishik Balerao, Ray J. Butcher

**Affiliations:** a Department of Chemistry, Yuvaraja’s College, University of Mysore, Mysore-570 005, India; b Department of Studies in Chemistry, University of Mysore, Manasagangotri, Mysore-570 006, India; c Department of Physics, Faculty of Sciences, Erciyes University, 38039, Kayseri, Türkiye; d Institute of Materials Science, Darmstadt University of Technology, Alarich-Weiss-Strasse 2, D-64287 Darmstadt, Germany; e Thomas Jefferson High School for Science and Technology, 6560 Braddock Rd, Alexandria VA 22312, USA; f Department of Chemistry, Howard University, 525 College Street NW, Washington DC 20059, USA; University of Aberdeen, United Kingdom

**Keywords:** crystal structure, piperazinium cation, carboxyl­ate anion, supra­molecular features, Hirshfeld analysis

## Abstract

The crystal structures and Hirshfeld surface analyses of three salts of 1-(4-nitro­phenyl)­piperazine with 2-chloro­benzoic acid, 2-bromo­benzoic acid and 2-iodo­benzoic acid are reported. The chloro­benzoate salt, C_10_H_14_N_3_O_2_^+^·C_7_H_4_ClO_2_^−^, contains whole-ion-disordered cations and anions, which were modeled with two equivalent conformations with occupancies of 0.745 (10)/0.255 (10) and 0.563 (13)/0.437 (13), respectively. The bromo­benzoate and iodo­benzoate derivatives are isomorphous and crystallize as hemihydrates, *viz*. C_10_H_14_N_3_O_2_^+^·C_7_H_4_BrO_2_^−^·0.5H_2_O and C_10_H_14_N_3_O_2_^+^·C_7_H_4_IO_2_^−^·0.5H_2_O, respectively [the water mol­ecule is disordered over two locations with occupancies of 0.276 (3)/0.223 (3) for the iodo­benzoate derivative]. In the extended structures, all three salts feature an *R* ^4^_4_(12) loop of two anions and two cations linked by N—H⋯O hydrogen bonds.

## Abstract 2

The crystal structures and Hirshfeld surface analyses of three salts of 1-(4-nitro­phenyl)­piperazine with 2-chloro­benzoic acid, 2-bromo­benzoic acid and 2-iodo­benzoic acid are reported.

## 1. Chemical context

Piperazines and substituted piperazines are pharmacophores that can be found in many biologically active compounds across a number of different therapeutic areas (Berkheij, 2005 ▸) such as anti­fungal (Upadhayaya *et al.*, 2004 ▸), anti-bacterial, anti-malarial and anti-psychotic agents (Chaudhary *et al.*, 2006 ▸). A review on the current pharmacological and toxicological information for piperazine derivatives was described (Elliott, 2011 ▸). 4-(4-Nitrophenyl)piperazin-1-ium chloride monohydrate has been used as an inter­mediate in the synthesis of anti­cancer drugs, transcriptase inhibitors and anti­fungal reagents and is also an important reagent for potassium channel openers, which show considerable biomolecular current-voltage rectification characteristics (Lu, 2007 ▸). 4-Nitro­phenyl­piperazine was the starting material in the synthesis and biological evaluation of piperazine containing hydrazone derivatives (Kaya *et al.*, 2016 ▸).

Very recently, we have reported the syntheses, crystal structures and Hirshfeld surface analysis of 4-(4-nitro­phen­yl)piperazin-1-ium tri­fluoro­acetate (Cambridge Structural Database refcode BEYREG) and 4-(4-nitro­phen­yl)piperazin-1-ium tri­chloro­acetate (BEYRIK) (Shankara Prasad *et al.*, 2023 ▸). As part of our ongoing studies in this area, the present paper reports the crystal structure studies and Hirshfeld surface analysis of three salts of 1-(4-nitro­phenyl)­piperazine with organic acids *viz*., 4-(4-nitrophenyl)piperazin-1-ium 2-chloro­benzoate, C_10_H_14_N_3_O_2_^+^·C_7_H_4_ClO_2_^−^ (**1**), 4-(4-nitrophenyl)piperazin-1-ium 2-bromo­benzoate hemihydrate, C_10_H_14_N_3_O_2_^+^·C_7_H_4_BrO_2_^−^·0.5H_2_O (**2**), and 4-(4-nitrophenyl)piperazin-1-ium 2-iodo­benzoate hemihydrate, C_10_H_14_N_3_O_2_^+^·C_7_H_4_IO_2_^−^·0.5H_2_O (**3**). 
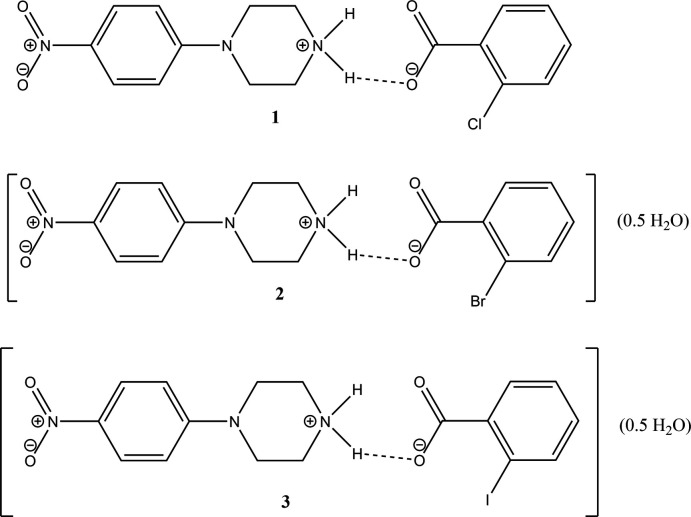


## 2. Structural commentary

**Figure 1:**
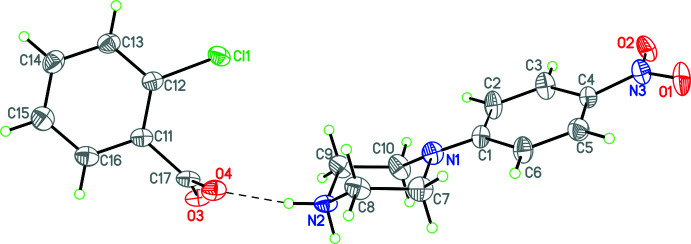
The mol­ecular structure of **1** with the N—H⋯O hydrogen bond shown as a dashed line. Atomic displacement parameters are at the 30% probability level.

Structure **1** consists of a 4-nitro­piperazinium cation linked to a 2-chloro­benzoate anion by two N—H⋯O hydrogen bonds (Fig. 1 ▸, Table 1 ▸), which will be discussed in further detail in the *Supra­molecular features* section of the paper. Both the cation and the anion exhibit whole-ion disorder, which was modeled with two equivalent conformations with occupancies of 0.745 (10)/0.255 (10) and 0.563 (13)/0.437 (13) respectively. When discussing the conformations of the anion and cation, only the major components will be used. In the chloro­benzoate anion, the carboxyl­ate group is significantly twisted with respect to the 2-chloro­phenyl ring with a dihedral angle of 76.7 (4)°, which is likely due to the steric inter­action between the *ortho*-chloro substituent and the carboxyl­ate group. Structures **2** and **3** exhibit similar cation conformations, with equivalent dihedral angles of 65.5 (3) and 67.1 (5)°, respectively. Additionally, in all three structures, the 4-nitro­phenyl group occupies an equatorial position in its attachment to the piperazinium ring.

**Figure 2:**
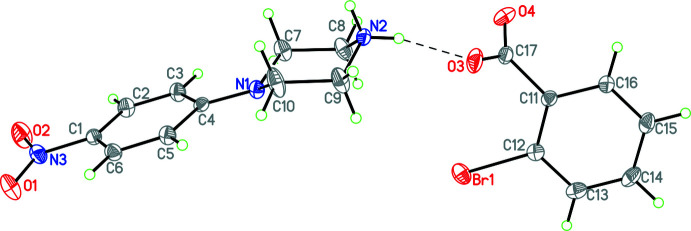
The mol­ecular structure of **2** with the N—H⋯O hydrogen bond shown as a dashed line. Atomic displacement parameters are at the 30% probability level.

Since **2** and **3** are isostructural, only **2** will be discussed in detail. This structure consists of a 4-(4-nitrophenyl)piperazin-1-ium cation linked to a 2-bromo­benzoate anion by two N—H⋯O hydrogen bonds (Figs. 2 ▸ and 3 ▸, Tables 2 ▸ and 3 ▸). Both **2** and **3** contain 0.5 water mol­ecules of crystallization [disordered over two locations with occupancies of 0.276 (3)/0.223 (3) for the iodo­benzoate derivative]. Additionally, there is a weak C—H⋯Br inter­action accepted by the bromine atom in the 2-bromo­bezoate anion and a carbon atom in the piperazinium ring, as well as a pair of weak C—H⋯O inter­actions between adjacent 4-nitro­phenyl rings in the 4-(4-nitrophenyl)piperazin-1-ium cation.

## 3. Supra­molecular features

**Figure 4:**
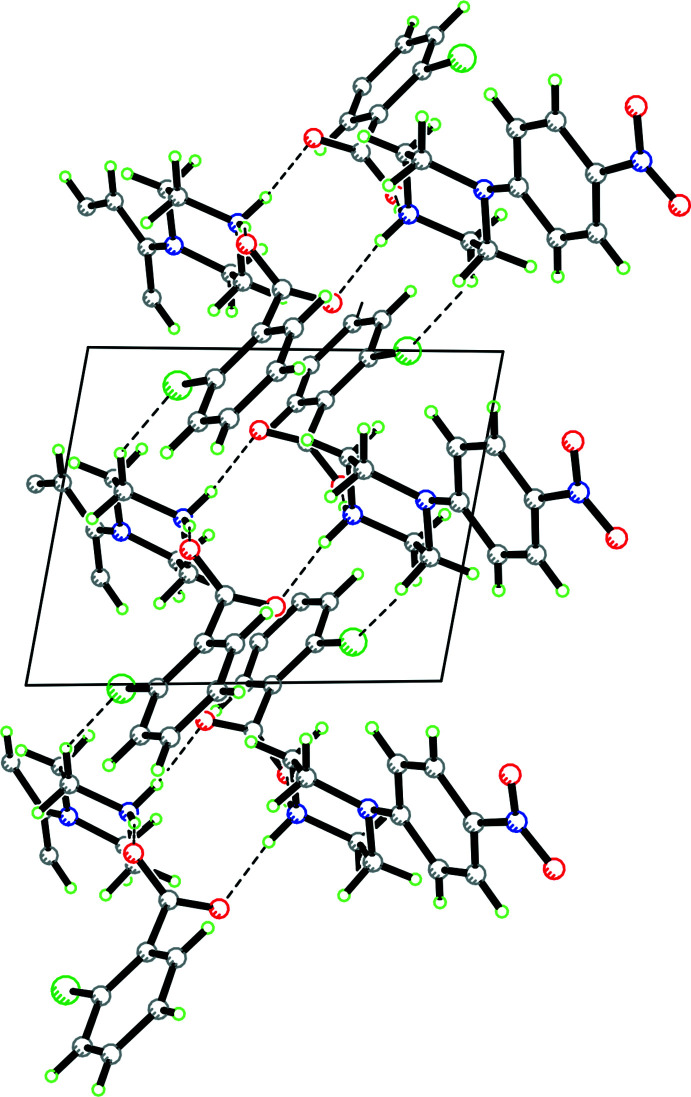
Packing diagram for **1** showing an 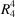 (12) loop of N—H⋯O hydrogen bonds with two cations and two anions (symmetry codes: 2 − *x*, 1 − *y*, 1 − *z*; *x*, 1 + *y*, *z*; 1 − *x*, 1 − *y*, 1 − *z*). Hydrogen bonds shown as dashed lines.

**Figure 5:**
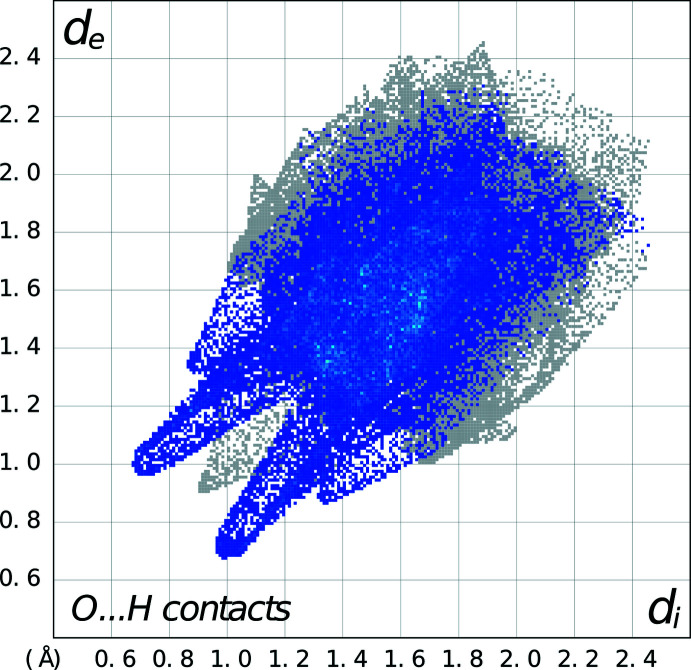
Fingerprint plot for **1** showing the N—H⋯O hydrogen bonds as prominent spikes.

In the packing of **1**, which contains both a disordered cation and anion as well as disordered water of solvation, the discussion will focus solely on the major component. The cation forms an 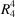 (12) loop involving N—H⋯O hydrogen bonds with two adjacent anions and an adjacent cation (symmetry codes: 2 − *x*, 1 − *y*, 1 − *z*; *x*, 1 + *y*, *z*; 1 − *x*, 1 − *y*, 1 − *z*; see Fig. 4 ▸ for packing and Fig. 5 ▸ for fingerprint plots). There is also a π–π inter­action between the nitro group in the cation and the phenyl ring of an adjacent cation [symmetry code: 1 − *x*, −*y*, −*z*; *Y*⋯*Cg* distance = 3.488 (18) Å; *X*—*Y*⋯*Cg*: 85.8 (12)°].

**Figure 6:**
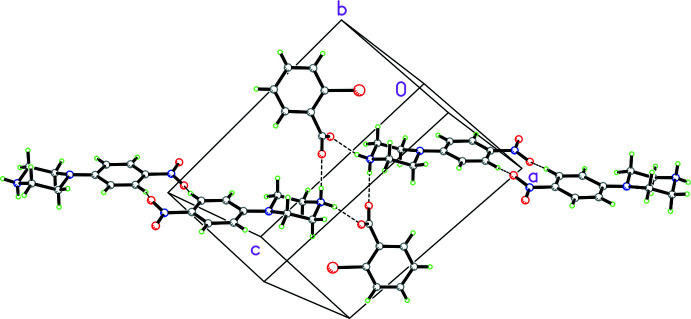
Packing diagram for **2** showing an 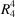 (12) loop arising from N—H⋯O hydrogen bonds with an adjacent cation and anion (symmetry code: 1 − *x*, −*y*, −*z*) and an 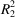 (10) loop comprised of weak C—H⋯O inter­actions between adjacent nitro­phenyl rings (symmetry code: −*x*, 1 − *y*, 1 − *z*). Hydrogen bonds and C—H⋯O inter­actions shown as dashed lines. The half occupancy water mol­ecule is omitted for clarity.

**Figure 7:**
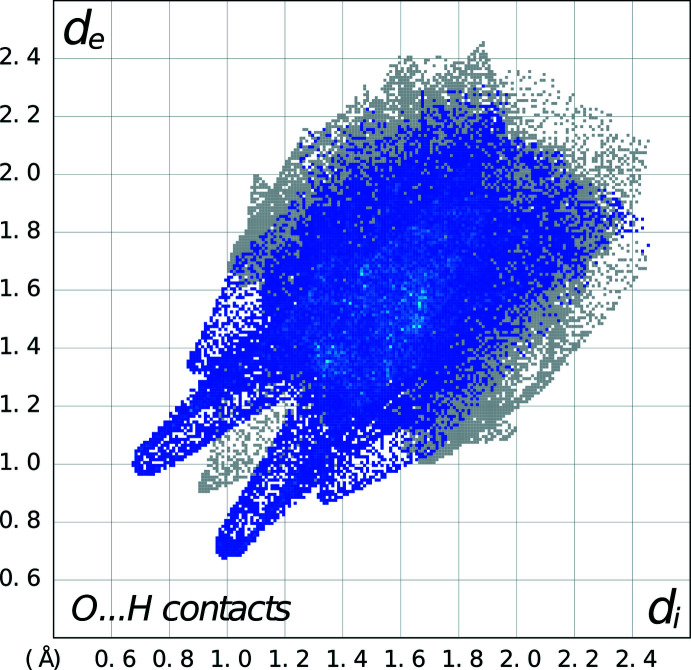
Fingerprint plot for **2** showing the N—H⋯O hydrogen bonds as prominent spikes.

In the packing of **2**, two cations and two anions form an 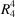 (12) loop (Etter *et al.*, 1990 ▸) of N—H⋯O hydrogen bonds (symmetry code: 1 − *x*, −*y*, −*z*; see Fig. 6 ▸ for packing and Fig. 7 ▸ for fingerprint plots). Additionally, there are weak C—H⋯O inter­actions between adjacent nitro­phenyl rings (symmetry code: −*x*, 1 − *y*, 1 − *z*) that form an 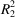 (10) ring (Fig. 6 ▸), as well as a weak C—H⋯Br inter­action between the piperazine ring and the bromine atom in an adjacent 2-bromo­benzoate anion (symmetry code: −1 + *x*, *y*, *z*). The phenyl rings in adjacent cations form π–π inter­actions with a perpendicular distance between centroids of 3.5332 (11) Å (symmetry code: 1 − *x*, 1 − *y*, 1 − *z*; slippage = 0.737 Å). These are all clearly seen in the fingerprint plot generated by *CrystalExplorer* (Spackman *et al.*, 2021 ▸).

**Figure 8:**
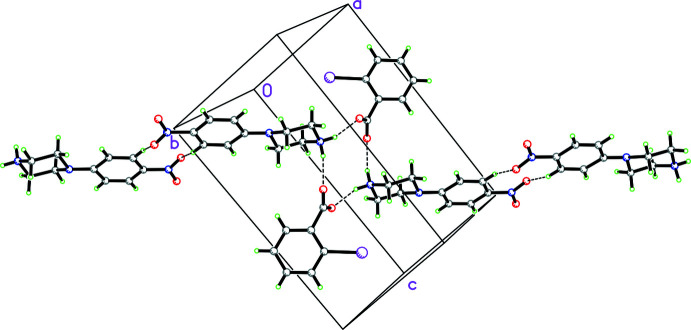
Packing diagram for **3** showing the same features as Fig. 6 ▸.

**Figure 9:**
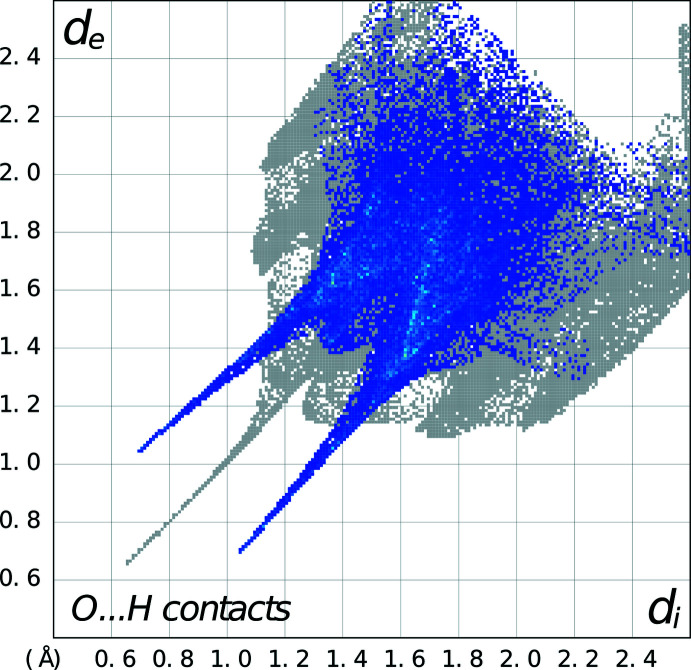
Fingerprint plot for **3** showing the N—H⋯O hydrogen bonding as prominent spikes.

In the packing of **3**, a pair of cations and a pair of anions form an 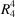 (12) loop linked by N—H⋯O hydrogen bonds (symmetry code: 1 − *x*, 1 − *y*, 1 − *z*; see Fig. 8 ▸ for packing and Fig. 9 ▸ for fingerprint plots). Additionally, there are weak C—H⋯O inter­actions between adjacent nitro­phenyl rings (symmetry code: 1 − *x*, 1 − *y*, −*z*) that form an 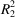 (10) ring. This structure contains a partially occupied water mol­ecule close to a center of inversion for which the hydrogen atoms were not able to be located (see *Refinement*). This species is likely to be involved in hydrogen bonding with an adjacent oxygen atom in the anion (symmetry code: 1 − *x*, 1 − *y*, 1 − *z*) and with the piperazine ring in the cation, forming an 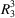 (10) ring. The phenyl rings in adjacent cations form π–π inter­actions with a perpendicular distance between centroids of 3.586 (4) Å (symmetry code: −*x*, 1 − *y*, −*z*; slippage = 0.379 Å).

## 4. Database survey

Related structures containing the 4-(4-nitrophenyl)piperazin-1-ium cation include 4-(4-nitrophenyl)piperazin-1-ium chloride monohydrate (refcode LIJNAU; Lu, 2007 ▸) and 4,6-dimeth­oxy pyrimidin-2-amine-1-(4-nitro­phen­yl)piperazine (1:1) (LUD­MUU; Wang *et al.*, 2014 ▸). Very recently, we have reported the crystal structures of six salts of 1-(4-nitro­phenyl)­piperazine (NEBVOJ, NEBVUP, NEBWAW, NEBWEA, NEBWIE, NEBWOK; Mahesha *et al.*, 2022*a* ▸). The syntheses and crystal structures of 4-(4-nitro­phen­yl)piperazin-1-ium benzoate monohydrate (BEFGIG) and 4-(4-nitro­phen­yl)piperazin-1-ium 2-carb­oxy-4,6-di­nitro­phenolate (BEFGOM) have been reported (Shankara Prasad *et al.*, 2022 ▸). A survey of these published derivatives containing the 4-(4-nitrophenyl)piperazin-1-ium cation shows that the most common conformation adopted by the 4-nitro­phenyl substituent with respect to the six-membered piperazinium ring is equatorial (LUDMUU, NEBVOJ, NEBVUP, NEBWAW, NEBWEA, NEBWOK, BEFGIG and BEYRIK) with only three adopting the axial conformation (LUDMUU, BEFGOM, and BEYREG). One published structure contains two 4-nitro­phenyl cations, with one adopting an equatorial conformation and the other an axial conformation (Mahesha *et al.*, 2022*a* ▸).

## 5. Synthesis and crystallization

For the synthesis of salts (**1**)–(**3**), a solution of commercially available (from Sigma-Aldrich) 1-(4-nitro­phenyl)­piperazine (100 mg, 0.483 mmol) in methanol (10 ml) was mixed with equimolar solutions of the appropriate acids in methanol (10 ml) and ethyl acetate (10 ml), *viz*., 2-chloro­benzoic acid (76 mg, 0.483 mmol) for (**1**), 2-bromo­benzoic acid (97 mg, 0.483 mmol) for (**2**), and 2-iodo­benzoic acid (120 mg, 0.483 mmol) for (**3**), The resulting solutions were stirred for 15 minutes at room temperature and allowed to stand at the same temperature. X-ray quality crystals were formed on slow evaporation after one week for all compounds, where ethanol:ethyl­acetate (1:1) was used for crystallization. The melting points are 439–441 K (**1**), 443–445 K (**2**) and 451–453 K (**3**).

## 6. Refinement

Crystal data, data collection and structure refinement details are summarized in Table 4 ▸. For all structures, the hydrogen atoms were located in difference maps and relocated to idealized locations (C—H = 0.93–0.97 Å) and refined as riding with *U*_iso_(H) = 1.2*U*_eq_(C) while the N—H hydrogen atoms were refined isotropically. For **1**, in which both the cation and the anion exhibit whole-ion disorder, two equivalent conformations were modeled with occupancies of 0.745 (10)/0.255 (10) and 0.563 (13)/ 0.437 (13) respectively. The water hydrogen atoms were refined isotropically with idealized geometries.

## Supplementary Material

Crystal structure: contains datablock(s) 1, 2, 3. DOI: 10.1107/S205698902300302X/hb8051sup1.cif

Structure factors: contains datablock(s) 1. DOI: 10.1107/S205698902300302X/hb80511sup2.hkl

Structure factors: contains datablock(s) 2. DOI: 10.1107/S205698902300302X/hb80512sup3.hkl

Structure factors: contains datablock(s) 3. DOI: 10.1107/S205698902300302X/hb80513sup4.hkl

CCDC references: 2253382, 2253381, 2253380

Additional supporting information:  crystallographic information; 3D view; checkCIF report

## Figures and Tables

**Figure 3:**
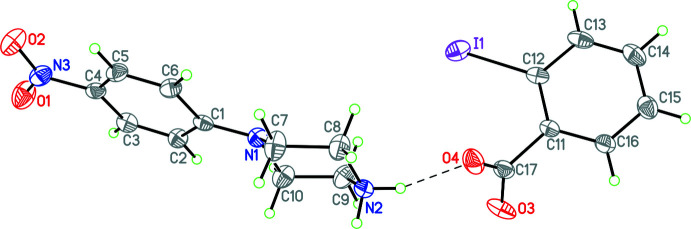
The mol­ecular structure of **3** with the N—H⋯O hydrogen bond shown as a dashed line. Atomic displacement parameters are at the 30% probability level.

**Table 1:** Hydrogen-bond geometry (Å, °) for (**1**)

*D*—H⋯*A*	*D*—H	H⋯*A*	*D*⋯*A*	*D*—H⋯*A*
C3—H3*A*⋯O2^i^	0.93	2.13	2.880 (15)	137
N2—H2*B*⋯O3^ii^	0.88 (2)	1.86 (2)	2.740 (6)	172 (3)
N2—H2*B*⋯O3*A* ^ii^	0.88 (2)	1.73 (2)	2.590 (13)	164 (3)
N2—H2*C*⋯O4	0.89 (2)	1.83 (2)	2.705 (8)	169 (3)
N2—H2*C*⋯O4*A*	0.89 (2)	1.81 (3)	2.644 (19)	156 (3)
C8—H8*A*⋯Cl1^iii^	0.97	2.82	3.629 (5)	142
C8—H8*A*⋯Cl1^iv^	0.97	2.95	3.780 (5)	144
C8—H8*A*⋯Cl1*A* ^iv^	0.97	2.88	3.643 (12)	136
C8—H8*B*⋯O4*A* ^iv^	0.97	2.58	3.13 (2)	116
C10—H10*A*⋯O3*A* ^ii^	0.97	2.65	3.285 (19)	123

Symmetry codes: (i) 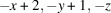 ; (ii) 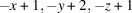 ; (iii) 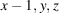 ; (iv) 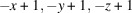 .

**Table 2:** Hydrogen-bond geometry (Å, °) for **2** [Chem scheme1]

*D*—H⋯*A*	*D*—H	H⋯*A*	*D*⋯*A*	*D*—H⋯*A*
N2—H2*A*⋯O3	0.85 (2)	1.83 (2)	2.655 (3)	164 (2)
N2—H2*B*⋯O4^i^	0.88 (2)	1.82 (2)	2.701 (3)	173 (2)
C2—H2⋯O2^ii^	0.93	2.50	3.307 (4)	145
C7—H7*B*⋯Br1^iii^	0.97	3.11	4.032 (2)	160
C10—H10*B*⋯O1*W* ^i^	0.97	2.10	3.057 (8)	169

Symmetry codes: (i) 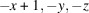 ; (ii) 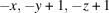 ; (iii) 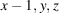 .

**Table 3:** Hydrogen-bond geometry (Å, °) for **3** [Chem scheme1]

*D*—H⋯*A*	*D*—H	H⋯*A*	*D*⋯*A*	*D*—H⋯*A*
C2—H2⋯I1^i^	0.93	3.28	4.110 (4)	150
C3—H3⋯O1^ii^	0.93	2.53	3.347 (7)	147
C6—H6⋯I1^iii^	0.93	3.26	3.940 (4)	132
C7—H7*A*⋯O1*WA*	0.97	2.14	3.09 (3)	167
C7—H7*A*⋯O1*WB*	0.97	2.01	2.85 (3)	145
N2—H2*A*⋯O3^iv^	0.88 (5)	1.86 (5)	2.717 (5)	164 (5)
N2—H2*B*⋯O4	0.93 (5)	1.77 (5)	2.666 (6)	160 (5)
O1*WB*—H1*W*3⋯O3^iv^	0.83 (2)	1.71 (10)	2.37 (2)	135 (12)

Symmetry codes: (i) 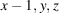 ; (ii) 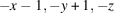 ; (iii) 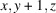 ; (iv) 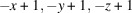 .

**Table 4:** Experimental details

	**1**	**2**	**3**
Crystal data			
Chemical formula	C_10_H_14_N_3_O_2_^+^·C_7_H_4_ClO_2_^−^	C_10_H_14_N_3_O_2_^+^·C_7_H_4_BrO_2_^−^·0.5H_2_O	C_10_H_14_N_3_O_2_^+^·C_7_H_4_IO_2_^−^·0.5H_2_O
*M*_r_	363.79	417.26	928.50
Crystal system, space group	Triclinic, *P* 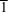	Triclinic, *P* 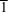	Triclinic, *P* 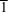
Temperature (K)	293	293	293
*a*, *b*, *c* (Å)	6.6073 (5), 8.2708 (5), 16.984 (1)	7.2570 (5), 9.7772 (6), 14.202 (1)	7.3949 (6), 9.3440 (8), 14.498 (1)
α, β, γ (°)	102.385 (6), 91.745 (6), 99.903 (6)	102.101 (6), 99.534 (6), 110.981 (6)	104.967 (8), 94.707 (7), 107.430 (8)
*V* (Å^3^)	890.84 (10)	887.41 (11)	909.44 (13)
*Z*	2	2	1
Radiation type	Mo *K*α	Mo *K*α	Mo *K*α
μ (mm^−1^)	0.24	2.35	1.79
Crystal size (mm)	0.48 × 0.44 × 0.24	0.36 × 0.32 × 0.20	0.50 × 0.44 × 0.24

Data collection			
Diffractometer	Oxford Diffraction Xcalibur CCD	Oxford Diffraction Xcalibur CCD	Oxford Diffraction Xcalibur CCD
Absorption correction	Multi-scan (*CrysAlis RED*; Oxford Diffraction, 2009 ▸)	Multi-scan (*CrysAlis RED*; Oxford Diffraction, 2009 ▸)	Multi-scan (*CrysAlis RED*; Oxford Diffraction, 2009 ▸)
*T*_min_, *T*_max_	0.894, 1.000	0.781, 1.000	0.697, 1.000
No. of measured, independent and observed [*I* > 2σ(*I*)] reflections	6637, 3787, 1916	6177, 3856, 2478	6275, 3904, 2443
*R*_int_	0.014	0.016	0.024
(sin θ/λ)_max_ (Å^−1^)	0.653	0.660	0.661

Refinement			
*R*[*F* ^2^ > 2σ(*F* ^2^)], *wR*(*F* ^2^), *S*	0.065, 0.162, 1.06	0.036, 0.084, 0.94	0.046, 0.117, 1.02
No. of reflections	3787	3856	3904
No. of parameters	357	244	264
No. of restraints	714	5	13
H-atom treatment	H atoms treated by a mixture of independent and constrained refinement	H atoms treated by a mixture of independent and constrained refinement	H atoms treated by a mixture of independent and constrained refinement
Δρ_max_, Δρ_min_ (e Å^−3^)	0.15, −0.15	0.51, −0.33	0.61, −0.44

Computer programs: *CrysAlis CCD* and *CrysAlis RED* (Oxford Diffraction, 2009 ▸), *SHELXL2018* (Sheldrick, 2015*b* ▸), *SHELXL2018/3* (Sheldrick, 2015*a* ▸) and *OLEX2* (Dolomanov *et al.*, 2009 ▸).

## supplementary crystallographic information

### 4-(4-Nitrophenyl)piperazin-1-ium 2-chlorobenzoate (1) Crystal data

**Table:**

C_10_H_14_N_3_O_2_^+^·C_7_H_4_ClO_2_^−^	*Z* = 2
*M**_r_* = 363.79	*F*(000) = 380
Triclinic, *P*1	*D*_x_ = 1.356 Mg m^−^^3^
*a* = 6.6073 (5) Å	Mo *K*α radiation, λ = 0.71073 Å
*b* = 8.2708 (5) Å	Cell parameters from 2365 reflections
*c* = 16.984 (1) Å	θ = 2.6–27.6°
α = 102.385 (6)°	µ = 0.24 mm^−^^1^
β = 91.745 (6)°	*T* = 293 K
γ = 99.903 (6)°	Prism, yellow
*V* = 890.84 (10) Å^3^	0.48 × 0.44 × 0.24 mm

### 4-(4-Nitrophenyl)piperazin-1-ium 2-chlorobenzoate (1) Data collection

**Table:**

Oxford Diffraction Xcalibur CCD diffractometer	1916 reflections with *I* > 2σ(*I*)
ω scans	*R*_int_ = 0.014
Absorption correction: multi-scan (CrysalisRed; Oxford Diffraction, 2009)	θ_max_ = 27.7°, θ_min_ = 2.6°
*T*_min_ = 0.894, *T*_max_ = 1.000	*h* = −8→8
6637 measured reflections	*k* = −10→7
3787 independent reflections	*l* = −20→21

### 4-(4-Nitrophenyl)piperazin-1-ium 2-chlorobenzoate (1) Refinement

**Table:**

Refinement on *F*^2^	Primary atom site location: dual
Least-squares matrix: full	Secondary atom site location: difference Fourier map
*R*[*F*^2^ > 2σ(*F*^2^)] = 0.065	Hydrogen site location: mixed
*wR*(*F*^2^) = 0.162	H atoms treated by a mixture of independent and constrained refinement
*S* = 1.06	*w* = 1/[σ^2^(*F*_o_^2^) + (0.0521*P*)^2^ + 0.3137*P*] where *P* = (*F*_o_^2^ + 2*F*_c_^2^)/3
3787 reflections	(Δ/σ)_max_ < 0.001
357 parameters	Δρ_max_ = 0.15 e Å^−^^3^
714 restraints	Δρ_min_ = −0.15 e Å^−^^3^

### 4-(4-Nitrophenyl)piperazin-1-ium 2-chlorobenzoate (1) Special details

**Table:**

Geometry. All esds (except the esd in the dihedral angle between two l.s. planes) are estimated using the full covariance matrix. The cell esds are taken into account individually in the estimation of esds in distances, angles and torsion angles; correlations between esds in cell parameters are only used when they are defined by crystal symmetry. An approximate (isotropic) treatment of cell esds is used for estimating esds involving l.s. planes.

### 4-(4-Nitrophenyl)piperazin-1-ium 2-chlorobenzoate (1) Fractional atomic coordinates and isotropic or equivalent isotropic displacement parameters (Å^2^)

**Table:**

	*x*	*y*	*z*	*U*_iso_*/*U*_eq_	Occ. (<1)
Cl1	1.0680 (5)	0.6989 (5)	0.5627 (2)	0.0833 (8)	0.745 (10)
O3	0.7965 (9)	1.0649 (9)	0.6034 (5)	0.0842 (18)	0.745 (10)
O4	0.6067 (11)	0.8100 (10)	0.5952 (5)	0.082 (2)	0.745 (10)
C11	0.9076 (7)	0.8971 (6)	0.6843 (2)	0.0615 (11)	0.745 (10)
C12	1.0545 (7)	0.7974 (6)	0.6616 (3)	0.0650 (12)	0.745 (10)
C13	1.1959 (7)	0.7776 (5)	0.7195 (3)	0.0835 (16)	0.745 (10)
H13	1.294232	0.710907	0.704298	0.100*	0.745 (10)
C14	1.1905 (8)	0.8575 (5)	0.8000 (3)	0.0943 (18)	0.745 (10)
H14	1.285124	0.844228	0.838711	0.113*	0.745 (10)
C15	1.0436 (9)	0.9572 (6)	0.8227 (2)	0.0931 (16)	0.745 (10)
H15	1.039942	1.010634	0.876538	0.112*	0.745 (10)
C16	0.9022 (8)	0.9770 (6)	0.7648 (3)	0.0776 (13)	0.745 (10)
H16	0.803866	1.043720	0.779953	0.093*	0.745 (10)
C17	0.7612 (19)	0.9300 (14)	0.6263 (9)	0.0666 (17)	0.745 (10)
Cl1A	1.0723 (17)	0.6949 (14)	0.5994 (9)	0.100 (3)	0.255 (10)
O3A	0.741 (3)	1.065 (3)	0.6227 (13)	0.082 (4)	0.255 (10)
O4A	0.654 (3)	0.785 (3)	0.5841 (13)	0.073 (4)	0.255 (10)
C11A	0.877 (2)	0.906 (2)	0.7016 (7)	0.066 (2)	0.255 (10)
C12A	1.030 (2)	0.8078 (18)	0.6923 (8)	0.069 (2)	0.255 (10)
C13A	1.1458 (19)	0.7951 (16)	0.7596 (10)	0.083 (2)	0.255 (10)
H13A	1.247604	0.729405	0.753378	0.099*	0.255 (10)
C14A	1.110 (2)	0.8807 (17)	0.8362 (8)	0.092 (3)	0.255 (10)
H14A	1.187433	0.872219	0.881231	0.111*	0.255 (10)
C15A	0.958 (2)	0.9790 (17)	0.8455 (7)	0.090 (3)	0.255 (10)
H15A	0.933424	1.036208	0.896755	0.108*	0.255 (10)
C16A	0.841 (2)	0.9916 (19)	0.7782 (8)	0.081 (2)	0.255 (10)
H16A	0.739583	1.057381	0.784426	0.097*	0.255 (10)
C17A	0.735 (7)	0.904 (5)	0.617 (3)	0.067 (3)	0.255 (10)
O1A	0.437 (2)	0.1420 (17)	−0.0678 (8)	0.143 (4)	0.437 (13)
O2A	0.725 (2)	0.2969 (18)	−0.0749 (8)	0.127 (3)	0.437 (13)
N3A	0.575 (2)	0.2519 (16)	−0.0385 (7)	0.105 (2)	0.437 (13)
C1A	0.548 (2)	0.5327 (19)	0.1991 (6)	0.071 (2)	0.437 (13)
C2A	0.713 (2)	0.5730 (16)	0.1536 (7)	0.085 (3)	0.437 (13)
H2	0.820154	0.661738	0.175667	0.102*	0.437 (13)
C3A	0.7186 (17)	0.4807 (15)	0.0753 (7)	0.089 (3)	0.437 (13)
H3	0.829021	0.507621	0.044865	0.107*	0.437 (13)
C4A	0.5589 (19)	0.3480 (14)	0.0424 (5)	0.086 (2)	0.437 (13)
C5A	0.3939 (18)	0.3077 (15)	0.0878 (7)	0.093 (2)	0.437 (13)
H5	0.287089	0.218996	0.065773	0.112*	0.437 (13)
C6A	0.389 (2)	0.4001 (18)	0.1662 (7)	0.089 (2)	0.437 (13)
H6	0.278218	0.373111	0.196576	0.107*	0.437 (13)
O1	0.5473 (18)	0.1306 (13)	−0.0808 (7)	0.144 (3)	0.563 (13)
O2	0.836 (2)	0.2893 (15)	−0.0755 (6)	0.161 (4)	0.563 (13)
N3	0.675 (2)	0.2455 (14)	−0.0465 (6)	0.109 (3)	0.563 (13)
C1	0.5784 (18)	0.5063 (14)	0.1894 (4)	0.070 (2)	0.563 (13)
C2	0.7641 (16)	0.5504 (13)	0.1562 (5)	0.089 (3)	0.563 (13)
H2A	0.866018	0.635336	0.185813	0.107*	0.563 (13)
C3	0.7975 (15)	0.4676 (12)	0.0787 (5)	0.095 (2)	0.563 (13)
H3A	0.921692	0.497043	0.056517	0.114*	0.563 (13)
C4	0.6452 (15)	0.3406 (11)	0.0345 (4)	0.086 (2)	0.563 (13)
C5	0.4595 (14)	0.2966 (11)	0.0676 (5)	0.087 (2)	0.563 (13)
H5A	0.357591	0.211638	0.038029	0.105*	0.563 (13)
C6	0.4261 (15)	0.3794 (13)	0.1451 (5)	0.0822 (19)	0.563 (13)
H6A	0.301914	0.349929	0.167326	0.099*	0.563 (13)
N1	0.5532 (4)	0.5957 (3)	0.27470 (16)	0.0768 (8)	
N2	0.5064 (4)	0.7717 (3)	0.43581 (17)	0.0675 (7)	
H2B	0.417 (4)	0.834 (3)	0.4252 (17)	0.081*	
H2C	0.530 (5)	0.795 (4)	0.4892 (11)	0.081*	
C7	0.3683 (5)	0.5526 (4)	0.3162 (2)	0.0921 (11)	
H7A	0.313237	0.433276	0.297924	0.110*	
H7B	0.264769	0.614262	0.302794	0.110*	
C8	0.4136 (5)	0.5935 (3)	0.4054 (2)	0.0858 (10)	
H8A	0.286923	0.568239	0.431384	0.103*	
H8B	0.506956	0.523703	0.419185	0.103*	
C9	0.6955 (5)	0.8126 (4)	0.3947 (2)	0.0899 (11)	
H9A	0.796744	0.749561	0.408843	0.108*	
H9B	0.752465	0.931538	0.412976	0.108*	
C10	0.6532 (6)	0.7714 (4)	0.3053 (2)	0.0929 (11)	
H10A	0.565368	0.844595	0.290746	0.111*	
H10B	0.781839	0.792344	0.279945	0.111*	

### 4-(4-Nitrophenyl)piperazin-1-ium 2-chlorobenzoate (1) Atomic displacement parameters (Å^2^)

**Table:**

	*U*^11^	*U*^22^	*U*^33^	*U*^12^	*U*^13^	*U*^23^
Cl1	0.0608 (8)	0.0760 (9)	0.0980 (18)	0.0096 (6)	0.0027 (12)	−0.0109 (13)
O3	0.056 (3)	0.069 (2)	0.139 (4)	0.019 (2)	0.014 (3)	0.041 (3)
O4	0.048 (3)	0.077 (3)	0.115 (4)	0.000 (2)	0.000 (3)	0.018 (2)
C11	0.053 (2)	0.0330 (18)	0.092 (3)	−0.0025 (16)	0.002 (2)	0.0088 (19)
C12	0.058 (2)	0.0400 (19)	0.088 (3)	0.0002 (16)	−0.009 (2)	0.003 (2)
C13	0.087 (3)	0.050 (2)	0.108 (4)	0.015 (2)	−0.016 (3)	0.007 (3)
C14	0.110 (4)	0.069 (3)	0.096 (4)	0.012 (3)	−0.029 (3)	0.009 (3)
C15	0.107 (4)	0.064 (3)	0.097 (3)	0.002 (3)	−0.004 (3)	0.004 (2)
C16	0.077 (3)	0.049 (2)	0.101 (3)	0.007 (2)	0.009 (2)	0.009 (2)
C17	0.047 (4)	0.048 (4)	0.101 (4)	0.007 (3)	0.010 (3)	0.009 (3)
Cl1A	0.071 (3)	0.079 (3)	0.140 (6)	0.017 (2)	0.020 (5)	−0.004 (5)
O3A	0.064 (8)	0.063 (5)	0.128 (9)	0.024 (6)	0.017 (7)	0.030 (6)
O4A	0.048 (8)	0.062 (6)	0.104 (7)	0.005 (6)	0.004 (6)	0.014 (5)
C11A	0.057 (4)	0.038 (4)	0.099 (4)	0.005 (3)	0.001 (4)	0.009 (4)
C12A	0.063 (4)	0.039 (4)	0.098 (4)	0.004 (3)	−0.007 (4)	0.009 (4)
C13A	0.085 (4)	0.054 (4)	0.103 (5)	0.011 (4)	−0.015 (4)	0.010 (4)
C14A	0.096 (5)	0.068 (5)	0.105 (5)	0.009 (5)	−0.010 (5)	0.009 (5)
C15A	0.089 (6)	0.066 (5)	0.106 (5)	0.001 (4)	0.000 (5)	0.011 (5)
C16A	0.080 (5)	0.054 (4)	0.101 (4)	0.001 (4)	0.002 (4)	0.008 (4)
C17A	0.051 (5)	0.047 (5)	0.102 (5)	0.011 (4)	0.006 (4)	0.015 (5)
O1A	0.145 (8)	0.134 (6)	0.107 (6)	−0.007 (6)	−0.031 (6)	−0.035 (5)
O2A	0.135 (7)	0.122 (6)	0.103 (6)	0.019 (6)	0.005 (6)	−0.019 (5)
N3A	0.117 (5)	0.090 (4)	0.089 (4)	0.007 (5)	−0.014 (4)	−0.007 (3)
C1A	0.090 (5)	0.033 (4)	0.080 (4)	0.002 (4)	−0.014 (3)	−0.001 (3)
C2A	0.095 (5)	0.053 (4)	0.091 (4)	−0.005 (4)	−0.014 (4)	−0.001 (3)
C3A	0.094 (5)	0.072 (4)	0.087 (4)	−0.001 (4)	−0.010 (4)	0.004 (3)
C4A	0.104 (5)	0.068 (3)	0.074 (3)	0.001 (4)	−0.018 (4)	−0.001 (3)
C5A	0.112 (5)	0.071 (4)	0.077 (5)	−0.009 (4)	−0.017 (4)	0.000 (4)
C6A	0.107 (5)	0.063 (4)	0.079 (5)	−0.006 (4)	−0.017 (4)	−0.001 (4)
O1	0.146 (7)	0.123 (4)	0.116 (5)	−0.016 (5)	−0.006 (5)	−0.043 (4)
O2	0.144 (7)	0.150 (5)	0.140 (5)	−0.011 (6)	0.036 (5)	−0.051 (4)
N3	0.125 (6)	0.090 (4)	0.088 (4)	−0.008 (5)	−0.003 (4)	−0.008 (3)
C1	0.090 (4)	0.033 (3)	0.082 (3)	0.007 (3)	−0.017 (3)	0.006 (3)
C2	0.099 (5)	0.059 (4)	0.092 (3)	−0.004 (4)	−0.003 (3)	−0.004 (3)
C3	0.103 (5)	0.074 (3)	0.090 (3)	−0.005 (4)	−0.005 (4)	−0.002 (3)
C4	0.105 (5)	0.068 (3)	0.076 (3)	0.004 (4)	−0.012 (3)	0.004 (2)
C5	0.108 (5)	0.068 (3)	0.069 (4)	−0.010 (3)	−0.015 (3)	0.002 (3)
C6	0.101 (4)	0.058 (3)	0.070 (4)	−0.011 (3)	−0.012 (3)	−0.001 (3)
N1	0.0767 (17)	0.0420 (12)	0.099 (2)	−0.0114 (11)	−0.0042 (15)	0.0076 (13)
N2	0.0543 (15)	0.0425 (12)	0.103 (2)	0.0079 (10)	0.0018 (15)	0.0101 (14)
C7	0.079 (2)	0.0508 (17)	0.127 (3)	−0.0174 (16)	−0.006 (2)	0.0043 (19)
C8	0.086 (2)	0.0425 (16)	0.121 (3)	−0.0017 (15)	0.014 (2)	0.0099 (17)
C9	0.061 (2)	0.074 (2)	0.111 (3)	−0.0155 (16)	0.0106 (19)	−0.0102 (19)
C10	0.096 (3)	0.0543 (18)	0.105 (3)	−0.0236 (17)	0.011 (2)	−0.0043 (18)

### 4-(4-Nitrophenyl)piperazin-1-ium 2-chlorobenzoate (1) Geometric parameters (Å, º)

**Table:**

Cl1—C12	1.715 (3)	C3A—C4A	1.3900
Cl1—H8A^i^	2.953 (5)	C3A—H3	0.9300
O3—C17	1.247 (14)	C4A—C5A	1.3900
O4—C17	1.304 (10)	C5A—C6A	1.3900
C11—C12	1.3900	C5A—H5	0.9300
C11—C16	1.3900	C6A—H6	0.9300
C11—C17	1.458 (15)	O1—N3	1.189 (9)
C12—C13	1.3900	O2—N3	1.216 (10)
C13—C14	1.3900	N3—C4	1.468 (8)
C13—H13	0.9300	C1—C2	1.3900
C14—C15	1.3900	C1—C6	1.3900
C14—H14	0.9300	C1—N1	1.507 (7)
C15—C16	1.3900	C2—C3	1.3900
C15—H15	0.9300	C2—H2A	0.9300
C16—H16	0.9300	C3—C4	1.3900
Cl1A—C12A	1.711 (4)	C3—H3A	0.9300
O3A—C17A	1.31 (4)	C4—C5	1.3900
O4A—C17A	1.06 (4)	C5—C6	1.3900
C11A—C12A	1.3900	C5—H5A	0.9300
C11A—C16A	1.3900	C6—H6A	0.9300
C11A—C17A	1.68 (4)	N1—C7	1.459 (4)
C12A—C13A	1.3900	N1—C10	1.464 (3)
C13A—C14A	1.3900	N2—C8	1.469 (3)
C13A—H13A	0.9300	N2—C9	1.474 (4)
C14A—C15A	1.3900	N2—H2B	0.882 (17)
C14A—H14A	0.9300	N2—H2C	0.889 (17)
C15A—C16A	1.3900	C7—C8	1.488 (5)
C15A—H15A	0.9300	C7—H7A	0.9700
C16A—H16A	0.9300	C7—H7B	0.9700
O1A—N3A	1.182 (10)	C8—H8A	0.9700
O2A—N3A	1.235 (12)	C8—H8B	0.9700
N3A—C4A	1.448 (9)	C9—C10	1.490 (5)
C1A—N1	1.275 (9)	C9—H9A	0.9700
C1A—C2A	1.3900	C9—H9B	0.9700
C1A—C6A	1.3900	C10—H10A	0.9700
C2A—C3A	1.3900	C10—H10B	0.9700
C2A—H2	0.9300		
			
C12—Cl1—H8A^i^	92.6 (2)	C4A—C5A—H5	120.0
C12—C11—C16	120.0	C5A—C6A—C1A	120.0
C12—C11—C17	122.9 (6)	C5A—C6A—H6	120.0
C16—C11—C17	117.0 (6)	C1A—C6A—H6	120.0
C11—C12—C13	120.0	O1—N3—O2	122.6 (9)
C11—C12—Cl1	121.34 (18)	O1—N3—C4	120.6 (8)
C13—C12—Cl1	118.66 (18)	O2—N3—C4	116.9 (7)
C14—C13—C12	120.0	C2—C1—C6	120.0
C14—C13—H13	120.0	C2—C1—N1	117.8 (6)
C12—C13—H13	120.0	C6—C1—N1	122.1 (6)
C13—C14—C15	120.0	C1—C2—C3	120.0
C13—C14—H14	120.0	C1—C2—H2A	120.0
C15—C14—H14	120.0	C3—C2—H2A	120.0
C14—C15—C16	120.0	C4—C3—C2	120.0
C14—C15—H15	120.0	C4—C3—H3A	120.0
C16—C15—H15	120.0	C2—C3—H3A	120.0
C15—C16—C11	120.0	C3—C4—C5	120.0
C15—C16—H16	120.0	C3—C4—N3	122.1 (5)
C11—C16—H16	120.0	C5—C4—N3	117.9 (5)
O3—C17—O4	122.2 (12)	C4—C5—C6	120.0
O3—C17—C11	119.3 (7)	C4—C5—H5A	120.0
O4—C17—C11	118.2 (10)	C6—C5—H5A	120.0
C12A—C11A—C16A	120.0	C5—C6—C1	120.0
C12A—C11A—C17A	116.6 (17)	C5—C6—H6A	120.0
C16A—C11A—C17A	123.3 (17)	C1—C6—H6A	120.0
C11A—C12A—C13A	120.0	C1A—N1—C7	117.5 (7)
C11A—C12A—Cl1A	121.3 (4)	C1A—N1—C10	118.6 (7)
C13A—C12A—Cl1A	118.7 (4)	C7—N1—C10	111.7 (3)
C14A—C13A—C12A	120.0	C7—N1—C1	121.9 (5)
C14A—C13A—H13A	120.0	C10—N1—C1	120.3 (5)
C12A—C13A—H13A	120.0	C8—N2—C9	109.7 (2)
C13A—C14A—C15A	120.0	C8—N2—H2B	108 (2)
C13A—C14A—H14A	120.0	C9—N2—H2B	109.3 (19)
C15A—C14A—H14A	120.0	C8—N2—H2C	111 (2)
C16A—C15A—C14A	120.0	C9—N2—H2C	112 (2)
C16A—C15A—H15A	120.0	H2B—N2—H2C	107 (3)
C14A—C15A—H15A	120.0	N1—C7—C8	111.2 (3)
C15A—C16A—C11A	120.0	N1—C7—H7A	109.4
C15A—C16A—H16A	120.0	C8—C7—H7A	109.4
C11A—C16A—H16A	120.0	N1—C7—H7B	109.4
O4A—C17A—O3A	140 (4)	C8—C7—H7B	109.4
O4A—C17A—C11A	118 (4)	H7A—C7—H7B	108.0
O3A—C17A—C11A	101 (2)	N2—C8—C7	111.6 (3)
O1A—N3A—O2A	123.5 (11)	N2—C8—H8A	109.3
O1A—N3A—C4A	118.5 (10)	C7—C8—H8A	109.3
O2A—N3A—C4A	117.8 (10)	N2—C8—H8B	109.3
N1—C1A—C2A	121.6 (9)	C7—C8—H8B	109.3
N1—C1A—C6A	117.4 (9)	H8A—C8—H8B	108.0
C2A—C1A—C6A	120.0	N2—C9—C10	111.3 (3)
C1A—C2A—C3A	120.0	N2—C9—H9A	109.4
C1A—C2A—H2	120.0	C10—C9—H9A	109.4
C3A—C2A—H2	120.0	N2—C9—H9B	109.4
C4A—C3A—C2A	120.0	C10—C9—H9B	109.4
C4A—C3A—H3	120.0	H9A—C9—H9B	108.0
C2A—C3A—H3	120.0	N1—C10—C9	111.9 (3)
C3A—C4A—C5A	120.0	N1—C10—H10A	109.2
C3A—C4A—N3A	118.0 (6)	C9—C10—H10A	109.2
C5A—C4A—N3A	122.0 (6)	N1—C10—H10B	109.2
C6A—C5A—C4A	120.0	C9—C10—H10B	109.2
C6A—C5A—H5	120.0	H10A—C10—H10B	107.9
			
C16—C11—C12—C13	0.0	O1A—N3A—C4A—C5A	4.2 (16)
C17—C11—C12—C13	177.0 (7)	O2A—N3A—C4A—C5A	179.5 (10)
C16—C11—C12—Cl1	−179.2 (4)	C3A—C4A—C5A—C6A	0.0
C17—C11—C12—Cl1	−2.3 (6)	N3A—C4A—C5A—C6A	177.9 (10)
H8A^i^—Cl1—C12—C11	−77.7	C4A—C5A—C6A—C1A	0.0
H8A^i^—Cl1—C12—C13	103.1	N1—C1A—C6A—C5A	−169.0 (13)
C11—C12—C13—C14	0.0	C2A—C1A—C6A—C5A	0.0
Cl1—C12—C13—C14	179.2 (4)	C6—C1—C2—C3	0.0
C12—C13—C14—C15	0.0	N1—C1—C2—C3	177.8 (9)
C13—C14—C15—C16	0.0	C1—C2—C3—C4	0.0
C14—C15—C16—C11	0.0	C2—C3—C4—C5	0.0
C12—C11—C16—C15	0.0	C2—C3—C4—N3	−177.9 (8)
C17—C11—C16—C15	−177.1 (6)	O1—N3—C4—C3	175.2 (11)
C12—C11—C17—O3	−99.0 (12)	O2—N3—C4—C3	−4.2 (14)
C16—C11—C17—O3	78.0 (14)	O1—N3—C4—C5	−2.8 (15)
C12—C11—C17—O4	75.1 (13)	O2—N3—C4—C5	177.8 (10)
C16—C11—C17—O4	−107.8 (11)	C3—C4—C5—C6	0.0
C16A—C11A—C12A—C13A	0.0	N3—C4—C5—C6	178.0 (8)
C17A—C11A—C12A—C13A	−176.2 (19)	C4—C5—C6—C1	0.0
C16A—C11A—C12A—Cl1A	177.7 (12)	C2—C1—C6—C5	0.0
C17A—C11A—C12A—Cl1A	1.5 (19)	N1—C1—C6—C5	−177.7 (9)
C11A—C12A—C13A—C14A	0.0	C2A—C1A—N1—C7	176.8 (6)
Cl1A—C12A—C13A—C14A	−177.7 (12)	C6A—C1A—N1—C7	−14.3 (11)
C12A—C13A—C14A—C15A	0.0	C2A—C1A—N1—C10	37.6 (12)
C13A—C14A—C15A—C16A	0.0	C6A—C1A—N1—C10	−153.6 (5)
C14A—C15A—C16A—C11A	0.0	C2—C1—N1—C7	−176.9 (4)
C12A—C11A—C16A—C15A	0.0	C6—C1—N1—C7	0.9 (9)
C17A—C11A—C16A—C15A	176 (2)	C2—C1—N1—C10	32.7 (8)
C12A—C11A—C17A—O4A	57 (5)	C6—C1—N1—C10	−149.5 (5)
C16A—C11A—C17A—O4A	−119 (4)	C1A—N1—C7—C8	164.1 (9)
C12A—C11A—C17A—O3A	−132 (2)	C10—N1—C7—C8	−54.0 (4)
C16A—C11A—C17A—O3A	52 (3)	C1—N1—C7—C8	153.4 (6)
N1—C1A—C2A—C3A	168.6 (14)	C9—N2—C8—C7	−57.1 (4)
C6A—C1A—C2A—C3A	0.0	N1—C7—C8—N2	56.4 (4)
C1A—C2A—C3A—C4A	0.0	C8—N2—C9—C10	56.2 (4)
C2A—C3A—C4A—C5A	0.0	C1A—N1—C10—C9	−164.9 (9)
C2A—C3A—C4A—N3A	−178.0 (10)	C7—N1—C10—C9	53.6 (4)
O1A—N3A—C4A—C3A	−177.9 (12)	C1—N1—C10—C9	−153.2 (6)
O2A—N3A—C4A—C3A	−2.6 (14)	N2—C9—C10—N1	−55.0 (4)

Symmetry code: (i) −*x*+1, −*y*+1, −*z*+1.

### 4-(4-Nitrophenyl)piperazin-1-ium 2-chlorobenzoate (1) Hydrogen-bond geometry (Å, º)

**Table:**

*D*—H···*A*	*D*—H	H···*A*	*D*···*A*	*D*—H···*A*
C3—H3*A*···O2^ii^	0.93	2.13	2.880 (15)	137
N2—H2*B*···O3^iii^	0.88 (2)	1.86 (2)	2.740 (6)	172 (3)
N2—H2*B*···O3*A*^iii^	0.88 (2)	1.73 (2)	2.590 (13)	164 (3)
N2—H2*C*···O4	0.89 (2)	1.83 (2)	2.705 (8)	169 (3)
N2—H2*C*···O4*A*	0.89 (2)	1.81 (3)	2.644 (19)	156 (3)
C8—H8*A*···Cl1^iv^	0.97	2.82	3.629 (5)	142
C8—H8*A*···Cl1^i^	0.97	2.95	3.780 (5)	144
C8—H8*A*···Cl1*A*^i^	0.97	2.88	3.643 (12)	136
C8—H8*B*···O4*A*^i^	0.97	2.58	3.13 (2)	116
C10—H10*A*···O3*A*^iii^	0.97	2.65	3.285 (19)	123

Symmetry codes: (i) −*x*+1, −*y*+1, −*z*+1; (ii) −*x*+2, −*y*+1, −*z*; (iii) −*x*+1, −*y*+2, −*z*+1; (iv) *x*−1, *y*, *z*.

### 4-(4-Nitrophenyl)piperazin-1-ium 2-bromobenzoate hemihydrate (2) Crystal data

**Table:**

C_10_H_14_N_3_O_2_^+^·C_7_H_4_BrO_2_^−^·0.5H_2_O	*Z* = 2
*M**_r_* = 417.26	*F*(000) = 426
Triclinic, *P*1	*D*_x_ = 1.562 Mg m^−^^3^
*a* = 7.2570 (5) Å	Mo *K*α radiation, λ = 0.71073 Å
*b* = 9.7772 (6) Å	Cell parameters from 2832 reflections
*c* = 14.202 (1) Å	θ = 3.0–27.9°
α = 102.101 (6)°	µ = 2.35 mm^−^^1^
β = 99.534 (6)°	*T* = 293 K
γ = 110.981 (6)°	Prism, brown
*V* = 887.41 (11) Å^3^	0.36 × 0.32 × 0.20 mm

### 4-(4-Nitrophenyl)piperazin-1-ium 2-bromobenzoate hemihydrate (2) Data collection

**Table:**

Oxford Diffraction Xcalibur CCD diffractometer	2478 reflections with *I* > 2σ(*I*)
ω scans	*R*_int_ = 0.016
Absorption correction: multi-scan (CrysalisRed; Oxford Diffraction, 2009)	θ_max_ = 28.0°, θ_min_ = 3.0°
*T*_min_ = 0.781, *T*_max_ = 1.000	*h* = −9→9
6177 measured reflections	*k* = −12→12
3856 independent reflections	*l* = −11→18

### 4-(4-Nitrophenyl)piperazin-1-ium 2-bromobenzoate hemihydrate (2) Refinement

**Table:**

Refinement on *F*^2^	Primary atom site location: dual
Least-squares matrix: full	Secondary atom site location: difference Fourier map
*R*[*F*^2^ > 2σ(*F*^2^)] = 0.036	Hydrogen site location: mixed
*wR*(*F*^2^) = 0.084	H atoms treated by a mixture of independent and constrained refinement
*S* = 0.94	*w* = 1/[σ^2^(*F*_o_^2^) + (0.0444*P*)^2^] where *P* = (*F*_o_^2^ + 2*F*_c_^2^)/3
3856 reflections	(Δ/σ)_max_ < 0.001
244 parameters	Δρ_max_ = 0.51 e Å^−^^3^
5 restraints	Δρ_min_ = −0.33 e Å^−^^3^

### 4-(4-Nitrophenyl)piperazin-1-ium 2-bromobenzoate hemihydrate (2) Special details

**Table:**

Geometry. All esds (except the esd in the dihedral angle between two l.s. planes) are estimated using the full covariance matrix. The cell esds are taken into account individually in the estimation of esds in distances, angles and torsion angles; correlations between esds in cell parameters are only used when they are defined by crystal symmetry. An approximate (isotropic) treatment of cell esds is used for estimating esds involving l.s. planes.

### 4-(4-Nitrophenyl)piperazin-1-ium 2-bromobenzoate hemihydrate (2) Fractional atomic coordinates and isotropic or equivalent isotropic displacement parameters (Å^2^)

**Table:**

	*x*	*y*	*z*	*U*_iso_*/*U*_eq_	Occ. (<1)
O1	0.4472 (3)	0.9088 (2)	0.57716 (16)	0.0866 (7)	
O2	0.1466 (4)	0.7360 (3)	0.53813 (19)	0.0965 (8)	
N1	0.5490 (3)	0.3809 (2)	0.27923 (14)	0.0452 (5)	
N2	0.6259 (3)	0.1873 (2)	0.12301 (15)	0.0462 (5)	
H2A	0.686 (3)	0.132 (3)	0.1015 (18)	0.055*	
H2B	0.556 (3)	0.198 (3)	0.0700 (14)	0.055*	
N3	0.3218 (4)	0.7818 (3)	0.53126 (16)	0.0593 (6)	
C1	0.3797 (4)	0.6788 (3)	0.46525 (17)	0.0447 (6)	
C2	0.2487 (4)	0.5276 (3)	0.4245 (2)	0.0540 (7)	
H2	0.123215	0.491207	0.439437	0.065*	
C3	0.3015 (4)	0.4304 (3)	0.36200 (19)	0.0518 (6)	
H3	0.209824	0.328530	0.333702	0.062*	
C4	0.4910 (3)	0.4806 (2)	0.33958 (16)	0.0399 (5)	
C5	0.6204 (4)	0.6357 (3)	0.38273 (19)	0.0493 (6)	
H5	0.746350	0.673689	0.368381	0.059*	
C6	0.5675 (4)	0.7326 (3)	0.44508 (18)	0.0505 (6)	
H6	0.657730	0.834750	0.473932	0.061*	
C7	0.3935 (4)	0.2306 (3)	0.2201 (2)	0.0611 (8)	
H7A	0.307731	0.241586	0.164666	0.073*	
H7B	0.307496	0.187484	0.261102	0.073*	
C8	0.4847 (5)	0.1232 (3)	0.1806 (2)	0.0676 (8)	
H8A	0.557431	0.102503	0.235884	0.081*	
H8B	0.376210	0.026977	0.138441	0.081*	
C9	0.7856 (4)	0.3349 (3)	0.1852 (2)	0.0781 (10)	
H9A	0.874756	0.378980	0.145729	0.094*	
H9B	0.867453	0.319121	0.239824	0.094*	
C10	0.6991 (5)	0.4438 (3)	0.2265 (3)	0.0808 (10)	
H10A	0.809491	0.536313	0.271886	0.097*	
H10B	0.635383	0.471694	0.172320	0.097*	
Br1	0.92610 (5)	−0.00832 (3)	0.31967 (2)	0.06679 (14)	
O3	0.8488 (3)	0.0263 (3)	0.09269 (16)	0.0782 (6)	
O4	0.6156 (3)	−0.2125 (3)	0.03302 (17)	0.0860 (7)	
C11	0.9379 (3)	−0.1685 (3)	0.12893 (18)	0.0438 (6)	
C12	1.0135 (4)	−0.1304 (3)	0.23087 (19)	0.0469 (6)	
C13	1.1536 (4)	−0.1827 (3)	0.2715 (2)	0.0609 (7)	
H13	1.201561	−0.157757	0.340436	0.073*	
C14	1.2204 (4)	−0.2701 (3)	0.2107 (3)	0.0671 (8)	
H14	1.316352	−0.303199	0.238116	0.080*	
C15	1.1471 (4)	−0.3102 (3)	0.1086 (2)	0.0630 (8)	
H15	1.193664	−0.369875	0.067067	0.076*	
C16	1.0048 (4)	−0.2615 (3)	0.0683 (2)	0.0545 (7)	
H16	0.952495	−0.291267	−0.000611	0.065*	
C17	0.7895 (5)	−0.1121 (4)	0.0817 (2)	0.0564 (7)	
O1W	0.5508 (9)	−0.5202 (7)	−0.0662 (6)	0.138 (2)	0.5
H1W1	0.435882	−0.430124	−0.060307	0.207*	0.5
H1W2	0.458306	−0.629327	−0.042348	0.207*	0.5

### 4-(4-Nitrophenyl)piperazin-1-ium 2-bromobenzoate hemihydrate (2) Atomic displacement parameters (Å^2^)

**Table:**

	*U*^11^	*U*^22^	*U*^33^	*U*^12^	*U*^13^	*U*^23^
O1	0.0975 (16)	0.0587 (13)	0.0842 (15)	0.0191 (13)	0.0368 (13)	−0.0055 (11)
O2	0.0777 (15)	0.0870 (16)	0.1195 (19)	0.0333 (13)	0.0502 (15)	0.0009 (14)
N1	0.0415 (11)	0.0324 (10)	0.0568 (12)	0.0109 (9)	0.0146 (10)	0.0093 (9)
N2	0.0471 (13)	0.0423 (12)	0.0490 (12)	0.0236 (10)	0.0069 (10)	0.0080 (10)
N3	0.0699 (17)	0.0574 (15)	0.0511 (13)	0.0261 (14)	0.0222 (13)	0.0119 (11)
C1	0.0523 (15)	0.0453 (14)	0.0398 (13)	0.0228 (13)	0.0121 (12)	0.0143 (11)
C2	0.0445 (14)	0.0516 (15)	0.0666 (17)	0.0165 (13)	0.0229 (13)	0.0177 (13)
C3	0.0435 (15)	0.0382 (13)	0.0670 (17)	0.0108 (12)	0.0159 (13)	0.0112 (12)
C4	0.0384 (13)	0.0357 (12)	0.0452 (13)	0.0148 (11)	0.0067 (11)	0.0149 (10)
C5	0.0413 (14)	0.0372 (13)	0.0630 (16)	0.0100 (11)	0.0163 (12)	0.0105 (12)
C6	0.0506 (15)	0.0378 (13)	0.0524 (15)	0.0121 (12)	0.0083 (13)	0.0066 (11)
C7	0.0520 (16)	0.0403 (14)	0.0762 (18)	0.0041 (13)	0.0293 (15)	0.0039 (13)
C8	0.088 (2)	0.0366 (14)	0.0735 (18)	0.0160 (15)	0.0382 (17)	0.0111 (13)
C9	0.0509 (17)	0.0548 (17)	0.101 (2)	0.0095 (14)	0.0262 (17)	−0.0155 (16)
C10	0.075 (2)	0.0370 (15)	0.125 (3)	0.0103 (14)	0.061 (2)	0.0078 (16)
Br1	0.0855 (2)	0.05620 (19)	0.05928 (19)	0.02352 (16)	0.03290 (16)	0.01687 (13)
O3	0.1037 (16)	0.0759 (15)	0.0967 (15)	0.0672 (14)	0.0419 (13)	0.0421 (12)
O4	0.0686 (14)	0.1081 (18)	0.0862 (15)	0.0493 (15)	−0.0021 (13)	0.0322 (14)
C11	0.0435 (13)	0.0383 (13)	0.0501 (14)	0.0172 (11)	0.0089 (12)	0.0154 (11)
C12	0.0479 (14)	0.0379 (13)	0.0538 (15)	0.0126 (12)	0.0135 (12)	0.0194 (11)
C13	0.0569 (17)	0.0535 (16)	0.0625 (17)	0.0141 (15)	0.0009 (15)	0.0236 (14)
C14	0.0530 (17)	0.0570 (18)	0.094 (2)	0.0271 (15)	0.0038 (17)	0.0318 (17)
C15	0.0583 (17)	0.0508 (16)	0.088 (2)	0.0320 (15)	0.0202 (16)	0.0180 (15)
C16	0.0564 (16)	0.0532 (16)	0.0554 (15)	0.0288 (14)	0.0108 (13)	0.0107 (13)
C17	0.067 (2)	0.076 (2)	0.0507 (15)	0.0494 (18)	0.0227 (15)	0.0258 (15)
O1W	0.118 (4)	0.096 (4)	0.197 (7)	0.036 (4)	0.044 (4)	0.046 (4)

### 4-(4-Nitrophenyl)piperazin-1-ium 2-bromobenzoate hemihydrate (2) Geometric parameters (Å, º)

**Table:**

O1—N3	1.204 (3)	C8—H8A	0.9700
O2—N3	1.216 (3)	C8—H8B	0.9700
N1—C4	1.387 (3)	C9—C10	1.487 (4)
N1—C10	1.452 (3)	C9—H9A	0.9700
N1—C7	1.456 (3)	C9—H9B	0.9700
N2—C9	1.461 (3)	C10—H10A	0.9700
N2—C8	1.465 (3)	C10—H10B	0.9700
N2—H2A	0.846 (16)	Br1—C12	1.896 (2)
N2—H2B	0.881 (16)	O3—C17	1.230 (3)
N3—C1	1.452 (3)	O4—C17	1.254 (3)
C1—C2	1.370 (3)	C11—C12	1.378 (3)
C1—C6	1.376 (3)	C11—C16	1.389 (3)
C2—C3	1.363 (3)	C11—C17	1.502 (4)
C2—H2	0.9300	C12—C13	1.389 (4)
C3—C4	1.400 (3)	C13—C14	1.355 (4)
C3—H3	0.9300	C13—H13	0.9300
C4—C5	1.399 (3)	C14—C15	1.377 (4)
C5—C6	1.361 (3)	C14—H14	0.9300
C5—H5	0.9300	C15—C16	1.375 (4)
C6—H6	0.9300	C15—H15	0.9300
C7—C8	1.492 (3)	C16—H16	0.9300
C7—H7A	0.9700	O1W—H1W1	1.4134
C7—H7B	0.9700	O1W—H1W2	1.1905
			
C4—N1—C10	118.22 (18)	C7—C8—H8A	109.4
C4—N1—C7	118.65 (17)	N2—C8—H8B	109.4
C10—N1—C7	112.0 (2)	C7—C8—H8B	109.4
C9—N2—C8	109.6 (2)	H8A—C8—H8B	108.0
C9—N2—H2A	106.7 (16)	N2—C9—C10	112.0 (2)
C8—N2—H2A	114.7 (17)	N2—C9—H9A	109.2
C9—N2—H2B	110.9 (17)	C10—C9—H9A	109.2
C8—N2—H2B	108.5 (16)	N2—C9—H9B	109.2
H2A—N2—H2B	106 (2)	C10—C9—H9B	109.2
O1—N3—O2	122.2 (2)	H9A—C9—H9B	107.9
O1—N3—C1	119.5 (2)	N1—C10—C9	112.8 (2)
O2—N3—C1	118.3 (2)	N1—C10—H10A	109.0
C2—C1—C6	120.1 (2)	C9—C10—H10A	109.0
C2—C1—N3	119.9 (2)	N1—C10—H10B	109.0
C6—C1—N3	120.0 (2)	C9—C10—H10B	109.0
C3—C2—C1	120.2 (2)	H10A—C10—H10B	107.8
C3—C2—H2	119.9	C12—C11—C16	118.0 (2)
C1—C2—H2	119.9	C12—C11—C17	122.6 (2)
C2—C3—C4	121.4 (2)	C16—C11—C17	119.3 (2)
C2—C3—H3	119.3	C11—C12—C13	120.8 (2)
C4—C3—H3	119.3	C11—C12—Br1	121.09 (19)
N1—C4—C5	121.73 (19)	C13—C12—Br1	118.1 (2)
N1—C4—C3	121.6 (2)	C14—C13—C12	120.0 (3)
C5—C4—C3	116.6 (2)	C14—C13—H13	120.0
C6—C5—C4	121.9 (2)	C12—C13—H13	120.0
C6—C5—H5	119.0	C13—C14—C15	120.4 (3)
C4—C5—H5	119.0	C13—C14—H14	119.8
C5—C6—C1	119.7 (2)	C15—C14—H14	119.8
C5—C6—H6	120.2	C16—C15—C14	119.7 (3)
C1—C6—H6	120.2	C16—C15—H15	120.2
N1—C7—C8	112.2 (2)	C14—C15—H15	120.2
N1—C7—H7A	109.2	C15—C16—C11	121.0 (3)
C8—C7—H7A	109.2	C15—C16—H16	119.5
N1—C7—H7B	109.2	C11—C16—H16	119.5
C8—C7—H7B	109.2	O3—C17—O4	126.0 (3)
H7A—C7—H7B	107.9	O3—C17—C11	117.9 (3)
N2—C8—C7	111.3 (2)	O4—C17—C11	116.2 (3)
N2—C8—H8A	109.4	H1W1—O1W—H1W2	105.4
			
O1—N3—C1—C2	170.2 (3)	N1—C7—C8—N2	−55.5 (3)
O2—N3—C1—C2	−9.8 (4)	C8—N2—C9—C10	−56.2 (3)
O1—N3—C1—C6	−8.7 (4)	C4—N1—C10—C9	165.9 (2)
O2—N3—C1—C6	171.2 (3)	C7—N1—C10—C9	−50.6 (4)
C6—C1—C2—C3	−1.4 (4)	N2—C9—C10—N1	53.6 (4)
N3—C1—C2—C3	179.7 (2)	C16—C11—C12—C13	0.5 (4)
C1—C2—C3—C4	1.4 (4)	C17—C11—C12—C13	−178.8 (2)
C10—N1—C4—C5	−26.0 (3)	C16—C11—C12—Br1	−178.41 (17)
C7—N1—C4—C5	−167.1 (2)	C17—C11—C12—Br1	2.4 (3)
C10—N1—C4—C3	155.3 (3)	C11—C12—C13—C14	1.2 (4)
C7—N1—C4—C3	14.2 (3)	Br1—C12—C13—C14	−179.9 (2)
C2—C3—C4—N1	177.4 (2)	C12—C13—C14—C15	−1.3 (4)
C2—C3—C4—C5	−1.3 (4)	C13—C14—C15—C16	−0.2 (4)
N1—C4—C5—C6	−177.4 (2)	C14—C15—C16—C11	1.9 (4)
C3—C4—C5—C6	1.4 (4)	C12—C11—C16—C15	−2.0 (4)
C4—C5—C6—C1	−1.4 (4)	C17—C11—C16—C15	177.2 (3)
C2—C1—C6—C5	1.4 (4)	C12—C11—C17—O3	65.2 (3)
N3—C1—C6—C5	−179.6 (2)	C16—C11—C17—O3	−114.1 (3)
C4—N1—C7—C8	−165.1 (2)	C12—C11—C17—O4	−115.8 (3)
C10—N1—C7—C8	51.5 (3)	C16—C11—C17—O4	64.9 (3)
C9—N2—C8—C7	57.1 (3)		

### 4-(4-Nitrophenyl)piperazin-1-ium 2-bromobenzoate hemihydrate (2) Hydrogen-bond geometry (Å, º)

**Table:**

*D*—H···*A*	*D*—H	H···*A*	*D*···*A*	*D*—H···*A*
N2—H2*A*···O3	0.85 (2)	1.83 (2)	2.655 (3)	164 (2)
N2—H2*B*···O4^i^	0.88 (2)	1.82 (2)	2.701 (3)	173 (2)
C2—H2···O2^ii^	0.93	2.50	3.307 (4)	145
C7—H7*B*···Br1^iii^	0.97	3.11	4.032 (2)	160
C10—H10*B*···O1*W*^i^	0.97	2.10	3.057 (8)	169

Symmetry codes: (i) −*x*+1, −*y*, −*z*; (ii) −*x*, −*y*+1, −*z*+1; (iii) *x*−1, *y*, *z*.

### 4-(4-Nitrophenyl)piperazin-1-ium 2-iodobenzoate hemihydrate (3) Crystal data

**Table:**

C_10_H_14_N_3_O_2_^+^·C_7_H_4_IO_2_^−^·0.5H_2_O	*Z* = 1
*M**_r_* = 928.50	*F*(000) = 462
Triclinic, *P*1	*D*_x_ = 1.695 Mg m^−^^3^
*a* = 7.3949 (6) Å	Mo *K*α radiation, λ = 0.71073 Å
*b* = 9.3440 (8) Å	Cell parameters from 2991 reflections
*c* = 14.498 (1) Å	θ = 3.0–28.0°
α = 104.967 (8)°	µ = 1.79 mm^−^^1^
β = 94.707 (7)°	*T* = 293 K
γ = 107.430 (8)°	Prism, orange
*V* = 909.44 (13) Å^3^	0.50 × 0.44 × 0.24 mm

### 4-(4-Nitrophenyl)piperazin-1-ium 2-iodobenzoate hemihydrate (3) Data collection

**Table:**

Oxford Diffraction Xcalibur CCD diffractometer	2443 reflections with *I* > 2σ(*I*)
ω scans	*R*_int_ = 0.024
Absorption correction: multi-scan (CrysalisRed; Oxford Diffraction, 2009)	θ_max_ = 28.0°, θ_min_ = 3.0°
*T*_min_ = 0.697, *T*_max_ = 1.000	*h* = −9→9
6275 measured reflections	*k* = −9→12
3904 independent reflections	*l* = −19→11

### 4-(4-Nitrophenyl)piperazin-1-ium 2-iodobenzoate hemihydrate (3) Refinement

**Table:**

Refinement on *F*^2^	Primary atom site location: dual
Least-squares matrix: full	Secondary atom site location: difference Fourier map
*R*[*F*^2^ > 2σ(*F*^2^)] = 0.046	Hydrogen site location: mixed
*wR*(*F*^2^) = 0.117	H atoms treated by a mixture of independent and constrained refinement
*S* = 1.02	*w* = 1/[σ^2^(*F*_o_^2^) + (0.0514*P*)^2^ + 0.5457*P*] where *P* = (*F*_o_^2^ + 2*F*_c_^2^)/3
3904 reflections	(Δ/σ)_max_ < 0.001
264 parameters	Δρ_max_ = 0.61 e Å^−^^3^
13 restraints	Δρ_min_ = −0.44 e Å^−^^3^

### 4-(4-Nitrophenyl)piperazin-1-ium 2-iodobenzoate hemihydrate (3) Special details

**Table:**

Geometry. All esds (except the esd in the dihedral angle between two l.s. planes) are estimated using the full covariance matrix. The cell esds are taken into account individually in the estimation of esds in distances, angles and torsion angles; correlations between esds in cell parameters are only used when they are defined by crystal symmetry. An approximate (isotropic) treatment of cell esds is used for estimating esds involving l.s. planes.

### 4-(4-Nitrophenyl)piperazin-1-ium 2-iodobenzoate hemihydrate (3) Fractional atomic coordinates and isotropic or equivalent isotropic displacement parameters (Å^2^)

**Table:**

	*x*	*y*	*z*	*U*_iso_*/*U*_eq_	Occ. (<1)
C1	0.1376 (6)	0.6393 (4)	0.1581 (3)	0.0555 (10)	
C2	−0.0631 (7)	0.5730 (5)	0.1440 (3)	0.0687 (12)	
H2	−0.116914	0.503974	0.178262	0.082*	
C3	−0.1822 (7)	0.6059 (6)	0.0822 (4)	0.0724 (13)	
H3	−0.314731	0.558330	0.073932	0.087*	
C4	−0.1064 (7)	0.7096 (5)	0.0318 (3)	0.0636 (11)	
C5	0.0897 (7)	0.7763 (6)	0.0429 (3)	0.0723 (13)	
H5	0.141500	0.845343	0.008346	0.087*	
C6	0.2093 (7)	0.7419 (5)	0.1044 (3)	0.0667 (12)	
H6	0.341659	0.787923	0.110643	0.080*	
C7	0.4418 (7)	0.7234 (7)	0.2667 (5)	0.0957 (18)	
H7A	0.418923	0.802683	0.317971	0.115*	
H7B	0.499045	0.773784	0.220487	0.115*	
C8	0.5801 (7)	0.6593 (7)	0.3092 (4)	0.0927 (17)	
H8A	0.619508	0.592894	0.257190	0.111*	
H8B	0.693988	0.745578	0.346016	0.111*	
C9	0.3145 (8)	0.4451 (6)	0.3207 (4)	0.0897 (16)	
H9A	0.256781	0.389355	0.364560	0.108*	
H9B	0.340842	0.370385	0.267916	0.108*	
C10	0.1766 (7)	0.5111 (6)	0.2811 (4)	0.0835 (15)	
H10A	0.061412	0.425824	0.244767	0.100*	
H10B	0.139797	0.577245	0.334373	0.100*	
C11	0.8424 (6)	0.2081 (5)	0.3805 (3)	0.0609 (11)	
C12	0.8206 (6)	0.1340 (5)	0.2821 (3)	0.0635 (12)	
C13	0.9344 (9)	0.0414 (6)	0.2499 (4)	0.0850 (16)	
H13	0.920205	−0.009287	0.184112	0.102*	
C14	1.0663 (9)	0.0254 (7)	0.3150 (5)	0.0919 (17)	
H14	1.143373	−0.034148	0.292932	0.110*	
C15	1.0857 (8)	0.0952 (7)	0.4112 (5)	0.0857 (15)	
H15	1.174200	0.082483	0.455149	0.103*	
C16	0.9743 (7)	0.1845 (6)	0.4432 (4)	0.0769 (13)	
H16	0.987579	0.231020	0.509488	0.092*	
C17	0.7295 (7)	0.3142 (7)	0.4194 (3)	0.0746 (13)	
I1	0.61864 (5)	0.15483 (4)	0.18127 (2)	0.08807 (17)	
N1	0.2589 (5)	0.6027 (4)	0.2184 (3)	0.0622 (9)	
N2	0.4954 (6)	0.5683 (5)	0.3724 (3)	0.0686 (10)	
H2A	0.482 (7)	0.635 (6)	0.424 (4)	0.082*	
H2B	0.596 (7)	0.539 (6)	0.396 (4)	0.082*	
N3	−0.2340 (7)	0.7474 (5)	−0.0316 (3)	0.0825 (12)	
O1	−0.4061 (7)	0.6959 (6)	−0.0342 (4)	0.1259 (16)	
O2	−0.1641 (7)	0.8273 (6)	−0.0819 (3)	0.1165 (14)	
O3	0.6139 (6)	0.2673 (6)	0.4715 (3)	0.1206 (15)	
O4	0.7641 (6)	0.4395 (5)	0.4000 (3)	0.0969 (11)	
O1WA	0.426 (5)	0.976 (3)	0.449 (2)	0.154 (7)	0.224 (3)
H1W1	0.396 (13)	1.048 (8)	0.483 (6)	0.231*	0.224 (3)
H1W2	0.470 (14)	1.011 (10)	0.406 (4)	0.231*	0.224 (3)
O1WB	0.507 (4)	0.935 (3)	0.4569 (19)	0.149 (6)	0.276 (3)
H1W3	0.427 (8)	0.892 (15)	0.486 (6)	0.224*	0.276 (3)
H1W4	0.604 (7)	0.916 (12)	0.475 (10)	0.224*	0.276 (3)

### 4-(4-Nitrophenyl)piperazin-1-ium 2-iodobenzoate hemihydrate (3) Atomic displacement parameters (Å^2^)

**Table:**

	*U*^11^	*U*^22^	*U*^33^	*U*^12^	*U*^13^	*U*^23^
C1	0.061 (2)	0.043 (2)	0.057 (2)	0.0145 (19)	0.023 (2)	0.0061 (18)
C2	0.068 (3)	0.062 (3)	0.076 (3)	0.014 (2)	0.020 (2)	0.027 (2)
C3	0.065 (3)	0.065 (3)	0.079 (3)	0.012 (2)	0.017 (2)	0.017 (2)
C4	0.078 (3)	0.057 (3)	0.049 (2)	0.021 (2)	0.015 (2)	0.0047 (19)
C5	0.083 (3)	0.071 (3)	0.063 (3)	0.017 (2)	0.026 (2)	0.026 (2)
C6	0.062 (3)	0.067 (3)	0.067 (3)	0.010 (2)	0.018 (2)	0.025 (2)
C7	0.067 (3)	0.082 (3)	0.140 (5)	0.007 (3)	0.005 (3)	0.062 (3)
C8	0.063 (3)	0.124 (4)	0.113 (4)	0.029 (3)	0.024 (3)	0.073 (4)
C9	0.098 (4)	0.081 (3)	0.091 (4)	0.015 (3)	0.013 (3)	0.047 (3)
C10	0.069 (3)	0.090 (4)	0.094 (3)	0.009 (3)	0.018 (3)	0.051 (3)
C11	0.066 (3)	0.056 (2)	0.063 (2)	0.022 (2)	0.024 (2)	0.016 (2)
C12	0.073 (3)	0.042 (2)	0.066 (3)	0.008 (2)	0.028 (2)	0.0089 (19)
C13	0.110 (4)	0.056 (3)	0.085 (3)	0.026 (3)	0.048 (3)	0.004 (2)
C14	0.097 (4)	0.073 (3)	0.125 (5)	0.044 (3)	0.051 (4)	0.035 (3)
C15	0.082 (3)	0.086 (4)	0.110 (4)	0.039 (3)	0.033 (3)	0.046 (3)
C16	0.085 (3)	0.089 (3)	0.072 (3)	0.040 (3)	0.031 (3)	0.031 (3)
C17	0.073 (3)	0.079 (3)	0.064 (3)	0.032 (3)	0.012 (2)	0.000 (2)
I1	0.0918 (3)	0.0799 (2)	0.0696 (2)	0.00239 (18)	0.00710 (17)	0.01674 (16)
N1	0.061 (2)	0.057 (2)	0.071 (2)	0.0143 (17)	0.0198 (18)	0.0276 (17)
N2	0.073 (2)	0.081 (3)	0.065 (2)	0.038 (2)	0.0256 (19)	0.025 (2)
N3	0.092 (3)	0.082 (3)	0.063 (2)	0.019 (2)	0.002 (2)	0.019 (2)
O1	0.091 (3)	0.159 (4)	0.138 (4)	0.035 (3)	0.005 (3)	0.075 (3)
O2	0.116 (3)	0.134 (4)	0.100 (3)	0.022 (3)	0.003 (2)	0.064 (3)
O3	0.119 (3)	0.124 (3)	0.130 (3)	0.058 (3)	0.075 (3)	0.019 (3)
O4	0.106 (3)	0.077 (2)	0.116 (3)	0.051 (2)	0.018 (2)	0.020 (2)
O1WA	0.208 (17)	0.155 (15)	0.160 (13)	0.119 (12)	0.070 (15)	0.070 (12)
O1WB	0.210 (16)	0.145 (13)	0.161 (12)	0.123 (11)	0.079 (14)	0.069 (11)

### 4-(4-Nitrophenyl)piperazin-1-ium 2-iodobenzoate hemihydrate (3) Geometric parameters (Å, º)

**Table:**

C1—N1	1.377 (5)	C10—H10A	0.9700
C1—C6	1.398 (6)	C10—H10B	0.9700
C1—C2	1.401 (6)	C11—C16	1.384 (7)
C2—C3	1.359 (7)	C11—C12	1.387 (6)
C2—H2	0.9300	C11—C17	1.511 (7)
C3—C4	1.376 (6)	C12—C13	1.403 (7)
C3—H3	0.9300	C12—I1	2.090 (5)
C4—C5	1.373 (6)	C13—C14	1.369 (8)
C4—N3	1.443 (7)	C13—H13	0.9300
C5—C6	1.367 (7)	C14—C15	1.354 (8)
C5—H5	0.9300	C14—H14	0.9300
C6—H6	0.9300	C15—C16	1.365 (7)
C7—N1	1.454 (6)	C15—H15	0.9300
C7—C8	1.496 (7)	C16—H16	0.9300
C7—H7A	0.9700	C17—O4	1.232 (6)
C7—H7B	0.9700	C17—O3	1.243 (6)
C8—N2	1.461 (6)	N2—H2A	0.88 (5)
C8—H8A	0.9700	N2—H2B	0.93 (5)
C8—H8B	0.9700	N3—O1	1.212 (6)
C9—N2	1.465 (7)	N3—O2	1.212 (6)
C9—C10	1.487 (7)	O1WA—H1W1	0.82 (2)
C9—H9A	0.9700	O1WA—H1W2	0.82 (2)
C9—H9B	0.9700	O1WB—H1W3	0.83 (2)
C10—N1	1.453 (5)	O1WB—H1W4	0.82 (2)
			
N1—C1—C6	121.4 (4)	N1—C10—H10B	109.3
N1—C1—C2	122.6 (4)	C9—C10—H10B	109.3
C6—C1—C2	115.9 (4)	H10A—C10—H10B	107.9
C3—C2—C1	122.4 (4)	C16—C11—C12	118.0 (4)
C3—C2—H2	118.8	C16—C11—C17	120.1 (4)
C1—C2—H2	118.8	C12—C11—C17	121.9 (4)
C2—C3—C4	120.0 (4)	C11—C12—C13	119.3 (5)
C2—C3—H3	120.0	C11—C12—I1	121.3 (3)
C4—C3—H3	120.0	C13—C12—I1	119.4 (4)
C5—C4—C3	119.5 (5)	C14—C13—C12	120.1 (5)
C5—C4—N3	120.9 (4)	C14—C13—H13	119.9
C3—C4—N3	119.6 (4)	C12—C13—H13	119.9
C6—C5—C4	120.5 (4)	C15—C14—C13	120.8 (5)
C6—C5—H5	119.8	C15—C14—H14	119.6
C4—C5—H5	119.8	C13—C14—H14	119.6
C5—C6—C1	121.7 (4)	C14—C15—C16	119.4 (6)
C5—C6—H6	119.2	C14—C15—H15	120.3
C1—C6—H6	119.2	C16—C15—H15	120.3
N1—C7—C8	112.7 (4)	C15—C16—C11	122.3 (5)
N1—C7—H7A	109.1	C15—C16—H16	118.9
C8—C7—H7A	109.1	C11—C16—H16	118.9
N1—C7—H7B	109.1	O4—C17—O3	126.3 (5)
C8—C7—H7B	109.1	O4—C17—C11	118.0 (5)
H7A—C7—H7B	107.8	O3—C17—C11	115.6 (5)
N2—C8—C7	111.8 (4)	C1—N1—C10	118.7 (4)
N2—C8—H8A	109.3	C1—N1—C7	117.8 (3)
C7—C8—H8A	109.3	C10—N1—C7	111.5 (4)
N2—C8—H8B	109.3	C8—N2—C9	110.3 (4)
C7—C8—H8B	109.3	C8—N2—H2A	107 (3)
H8A—C8—H8B	107.9	C9—N2—H2A	114 (3)
N2—C9—C10	111.7 (4)	C8—N2—H2B	104 (3)
N2—C9—H9A	109.3	C9—N2—H2B	119 (3)
C10—C9—H9A	109.3	H2A—N2—H2B	102 (5)
N2—C9—H9B	109.3	O1—N3—O2	122.4 (5)
C10—C9—H9B	109.3	O1—N3—C4	119.3 (5)
H9A—C9—H9B	107.9	O2—N3—C4	118.3 (5)
N1—C10—C9	111.8 (4)	H1W1—O1WA—H1W2	103 (3)
N1—C10—H10A	109.3	H1W3—O1WB—H1W4	103 (3)
C9—C10—H10A	109.3		
			
N1—C1—C2—C3	−178.0 (4)	C12—C11—C16—C15	1.9 (7)
C6—C1—C2—C3	−0.1 (6)	C17—C11—C16—C15	−177.1 (5)
C1—C2—C3—C4	−1.0 (7)	C16—C11—C17—O4	111.4 (5)
C2—C3—C4—C5	1.5 (7)	C12—C11—C17—O4	−67.5 (6)
C2—C3—C4—N3	−178.2 (4)	C16—C11—C17—O3	−66.2 (6)
C3—C4—C5—C6	−0.8 (7)	C12—C11—C17—O3	114.9 (5)
N3—C4—C5—C6	178.8 (4)	C6—C1—N1—C10	172.5 (4)
C4—C5—C6—C1	−0.3 (7)	C2—C1—N1—C10	−9.7 (6)
N1—C1—C6—C5	178.7 (4)	C6—C1—N1—C7	32.9 (6)
C2—C1—C6—C5	0.8 (6)	C2—C1—N1—C7	−149.3 (5)
N1—C7—C8—N2	−53.1 (7)	C9—C10—N1—C1	164.4 (4)
N2—C9—C10—N1	56.1 (6)	C9—C10—N1—C7	−53.6 (6)
C16—C11—C12—C13	−1.3 (6)	C8—C7—N1—C1	−165.4 (4)
C17—C11—C12—C13	177.7 (4)	C8—C7—N1—C10	52.3 (7)
C16—C11—C12—I1	177.8 (3)	C7—C8—N2—C9	54.3 (7)
C17—C11—C12—I1	−3.3 (6)	C10—C9—N2—C8	−56.0 (6)
C11—C12—C13—C14	−0.4 (7)	C5—C4—N3—O1	−174.4 (5)
I1—C12—C13—C14	−179.5 (4)	C3—C4—N3—O1	5.2 (7)
C12—C13—C14—C15	1.6 (8)	C5—C4—N3—O2	7.1 (7)
C13—C14—C15—C16	−1.0 (8)	C3—C4—N3—O2	−173.3 (5)
C14—C15—C16—C11	−0.7 (8)		

### 4-(4-Nitrophenyl)piperazin-1-ium 2-iodobenzoate hemihydrate (3) Hydrogen-bond geometry (Å, º)

**Table:**

*D*—H···*A*	*D*—H	H···*A*	*D*···*A*	*D*—H···*A*
C2—H2···I1^i^	0.93	3.28	4.110 (4)	150
C3—H3···O1^ii^	0.93	2.53	3.347 (7)	147
C6—H6···I1^iii^	0.93	3.26	3.940 (4)	132
C7—H7*A*···O1*WA*	0.97	2.14	3.09 (3)	167
C7—H7*A*···O1*WB*	0.97	2.01	2.85 (3)	145
N2—H2*A*···O3^iv^	0.88 (5)	1.86 (5)	2.717 (5)	164 (5)
N2—H2*B*···O4	0.93 (5)	1.77 (5)	2.666 (6)	160 (5)
O1*WB*—H1*W*3···O3^iv^	0.83 (2)	1.71 (10)	2.37 (2)	135 (12)

Symmetry codes: (i) *x*−1, *y*, *z*; (ii) −*x*−1, −*y*+1, −*z*; (iii) *x*, *y*+1, *z*; (iv) −*x*+1, −*y*+1, −*z*+1.
